# Relations between the EU, Turkey, and Japan: dissonances in the strategic triangle

**DOI:** 10.12688/f1000research.51085.1

**Published:** 2021-03-02

**Authors:** Atsuko Higashino

**Affiliations:** 1 Graduate School of Business Sciences, Humanities and Social Sciences, University of Tsukuba, Tsukuba-shi, Ibaraki, 305-0006, Japan

**Keywords:** European Union (EU), Turkey, Japan, Economic Partnership Agreement (EPA), Strategic Partnership Agreement (SPA), Customs Union, Accession, Connectivity

## Abstract

This paper considers how the three sets of bilateral relations, between the European Union (EU) and Japan, the EU and Turkey, and Turkey and Japan, have developed (or been underdeveloped) and how the three have failed to form a strategic triangle that could potentially be beneficial for dealing with regional and international problems more efficiently. One of the main arguments is that, although all three sides of this triangle have developed significant economic relationships, their political relationships are less consolidated. Such a phenomenon is largely illustrated by the following three elements of this triangle: a *deteriorated* relationship between the EU and Turkey, an *underutilised* relationship between Japan and the EU, and an *extant* relationship between Japan and Turkey. This paper analyses the elements that have impeded or continue to hinder constructive political dialogue. It concludes that the potential for improvement in the three sets of bilateral relations is slight, in the short term, with Japan, in particular, finding it increasingly difficult to strike a good balance between developing the relationship with the EU while maintaining historical ties with Turkey.

## Introduction

The early version of this paper was presented at the 6th biannual European Union in International Affairs conference in 2018 ( Higashino, 2018). On 8 December 2017, the EU and Japan finalised the EU–Japan Economic Partnership Agreement (EPA) after nearly five years of negotiations. The agreement was signed on 17 July 2018 and came into force on 1 February 2019, ahead of the original target date of 31 October 2019. Currently, the largest free trade agreement (FTA) in the world, which covers over 600 million people, the EU–Japan EPA, is set to have considerable global economic impact. The EU also concluded parallel negotiations on a Strategic Partnership Agreement (SPA) with Japan on 25 April 2018, which was signed on 17 July together with the aforementioned EPA. The SPA aims to reinforce the overall partnership between the two countries from a strategic perspective and to facilitate common solutions to shared challenges such as climate change, energy supply, and security threats.

The strengthening of the relationship between the EU and Japan has already affected their respective relationships with other countries. For instance, in light of the EU–Japan EPA negotiations and the EU–Turkey Customs Union that has been in place since 1995, the EPA between Japan and Turkey was negotiated in 2013. Stronger ties between the EU, Turkey, and Japan are, in principle, to be welcomed. Since all play leading roles in the G20, freer trade between them and closer consultation on various international affairs would enhance their respective positions as global actors, potentially enabling them to form an influential strategic triangle, in terms of politics, economy, and security. Such a triangle would be an important element for stability in the face of the uncertainties, such as those posed by the Trump administration in the US, as well as Brexit in the UK. As it stands, however, things are not developing in that direction. The relationship between the EU and Turkey has deteriorated, largely because of the significant democratic setbacks in Turkey over the past few years (e.g., Danforth, 2017; Rubin, 2017), such as oppression of freedom of speech, the press, and assembly. Mutual condemnation between the Erdoğan government and the EU is becoming ever harsher, and Turkey’s EU accession prospects are on the verge of collapse. Another side of the triangle does not seem to be promising either—the EU–Japan relationship requires consolidation before both sides can discuss some tricky and delicate issues facing them, including the democratic crisis in Turkey and how best to manage it together. Despite relations between Japan and Turkey rarely having had any problems, endeavours to substantiate their political and diplomatic relations remain largely undeveloped.

This paper first provides the background for the commencement of the Turkey–Japan EPA negotiations, explaining that it is a logical extension of the EU–Turkey Customs Union and the EU–Japan EPA negotiations. It then considers how the three sets of bilateral relations have developed (or been underdeveloped) and how the three have failed to form a strategic triangle. One of the main arguments is that all three sides of this triangle have developed strong economic relationships, but their political relationships are less consolidated. Such a phenomenon is largely illustrated by the following three elements of this triangle: first, the relationship between the EU and Turkey has deteriorated to an unprecedented level (referred to as *deteriorated* below); not only is the accession process at a standstill—at the time of writing the EU was considering sanctions over Turkey’s increasingly aggressive foreign policy in the East Mediterranean region—but the renewal of the Customs Union is also unlikely to materialise in the near future. Second, despite their long-standing endeavour to develop political dialogue, and despite repeated declarations that both sides share fundamental values and norms, the relationship between the EU and Japan has not reached a point of discussion and alignment over the trickiest problems facing them (referred to as *underutilised* below). Third, while historical ties and friendship have often been underscored, and recent endeavours to upgrade their economic relations have been significant, the relationship between Turkey and Japan is still dependent on the events and discourses of the past, falling far short of creating ties that enable the two countries to consult over more than bilateral issues (referred to as *extant* below).

There has been a rich quantity and quality of literature on the EU–Turkey relationship, a significant part of which focuses on the process of Turkey’s accession process to the EU and difficulties pertaining to it (e.g., Aydın-Düzgit & Tocci, 2015; Buzan & Diez, 1999; Pererini, 2017). With regard to the literature on the Japan–EU relationship, there has been a considerable shift in the tone assessing the relationship between the two sides, notably before the signing of the EPA/SPA and after. Although many studies used to problematise the rather inactive political relationship between the EU and Japan (e.g., Higashino, 2016; Tsuruoka, 2008), the emphasis of studies which followed the materialisation of the EPA/SPA has been on how to better substantiate and consolidate the relationship between the two parties, with more of an optimistic tone than in previous years (e.g., Heijmans, 2019; Söderberg, 2020; Tsuruoka, 2019; Tsuruoka, 2020). In terms of the Turkey–Japan relationship, although there have been some excellent overviews of the relations between the two countries ( Pehlivanturk, 2011-12; Yilmaz, 2012), the quantity of the research is less than the studies on EU–Turkey and EU–Japan relations, and it has rarely been built upon. However, as this paper emphasises, the three relationships have mutually affected one another and the strengthening and worsening of the relationship of one side of the triangle (e.g., EU–Turkey) inevitably affects the other two (e.g., EU–Japan and Japan–EU). Such intertwining has not been explicitly discussed in previous research. One of the main purposes of this paper is therefore to explore and discuss the interconnectedness of the three sets of relationships. It should be noted that the strengthening of one side of this EU–Japan–Turkey relationship does not necessarily lead to positive implications on the other sides; as this paper shows, for example, strengthening the EU–Japan relationship via its EPA could potentially have negative impacts on Turkey’s trade with other countries, if the endeavours to modernise the Customs Union between the EU and Turkey are not realised.

In addition to the analyses mentioned above, this paper considers a potential implication of the deteriorating relationship of one side of the triangle—the EU and Turkey—shedding light on Turkey’s recent *rapprochement* with non-Western organisations and frameworks, *inter alia*, the Shanghai Cooperation Organisation (SCO). Erdoğan’s repeated references to Turkey’s intention to seek formal membership in the SCO has raised concerns both from the EU and, to a lesser extent, from Japan, as it has largely been seen as an indication that Turkey is moving farther away from the Western community and closer to a Russia-China-led regional/international order.

## Background: bilateral trade agreements

The current Turkey–Japan EPA negotiation is a logical extension of two important trade agreements: The Turkey–EU Customs Union came into force in 1996 and the EU–Japan EPA came into force in 2019. Turkey’s relations with the EU are based on the Ankara Agreement establishing an Association between the European Economic Community (EEC) and Turkey, thus creating the Customs Union between the two. EU Customs Union Decision 1/95 requires Turkey to align its commercial policy with the EU’s Common Commercial Policy, which includes, *inter alia*, the EU’s preferential trade agreements ( Official Journal of the European Communities, 1996). In this context, Turkey has been expected to negotiate agreements on a mutually advantageous basis with other countries with which the EU already has an FTA ( Joint Study Group, 2013: 4). This means that Turkey and the EU should have ‘parallel FTAs in force’ ( Joint Study Group, 2013: 4). To this end, Turkey has initiated a negotiation process and concluded FTAs with third countries at almost the same time as the EU ( Joint Study Group 2013: 5).

Therefore, the commencement of EPA/SPA negotiations between the EU and Japan in March 2013 necessitated FTA negotiations between Turkey and Japan. At the G20 Summit in November 2011, Turkish President Recep Tayyip Erdoğan told the then Prime Minister Yoshihiko Noda of Japan that he expected an EPA/SPA agreement to be negotiated between Japan and Turkey. After almost a month, in December 2011, Turkish Deputy Prime Minister, Ali Babacan, expressed Turkey’s intention to commence negotiations for a Japan–Turkey EPA/SPA in parallel with the EU–Japan EPA negotiations In July 2012, both sides agreed to launch a Joint Study Group for a Turkey–Japan EPA. Following the publication of the Report of the Joint Study Group in July 2013, formal negotiations began in December 2014. As of January 2021, 17 rounds of negotiations had been conducted (no negotiation tool place in 2020). However, as will be discussed below, such developments on each side of the EU–Turkey–Japan triangle in trade agreements have not led to the construction of more open, solid, and constructive political relationships between the three. The following sections of this paper will introduce the respective problems that characterise each relationship.

## EU–Turkey relationship: renewed Customs Union for a lesser evil

Since it has already been widely understood that Turkey’s accession to the EU is not likely to happen for years to come ( Blockmans & Yilmaz, 2017; Wagstyl & Chazan, 2017), one of the few options that could reinforce the relationship between the EU and Turkey is the renewal of the existing EU–Turkey Customs Union. As it stands, it is a rather limited agreement that covers all industrial goods and processed agricultural products. Still, it has contributed to the expansion of trade between the EU and Turkey; in 2019, Turkey was the EU’s 5 ^th^-largest trading partner, export market, and provider of imports, while the EU is Turkey’s most important trading partner ( European Commission, 2020a). However, it is a trade agreement concluded 20 years ago and without a doubt needs an overhaul: the 1995 Customs Union was intended to be ‘an interim process, and not an end in itself’ ( Vesterbye & Akman, 2017: 5).

In recent times, Turkey has been seriously affected by the conclusions of the several FTA/EPA by the EU with third countries. Turkey is expected to grant tariff-free access to goods from a third country with which the EU has negotiated an FTA, without receiving any reciprocal access. Further, Turkey does not have any influence over when the EU negotiates an FTA/EPA with third countries. Unless Turkey manages to conclude a similar deal with a third country, it could be forced to suffer unilaterally from a disadvantageous trade position. For instance, when the Transatlantic Trade and Investment Partnership (TTIP) was negotiated between the US and the EU before the inauguration of the Trump administration, Turkey was concerned that the TTIP could create discrimination against Turkey since the country could well have been forced to adjust its external tariffs to the US according to the rules of the Customs Union, while Turkey was not expected to have an equal share in the benefits stemming from the agreement ( Yalcin *et al.*, 2016). While the TTIP negotiations have been halted since early 2017, such concerns could well be raised whenever the EU negotiates to conclude new trade deals with third countries. Such a situation has already occurred in the case of the EU–Mexico EPA, in which Mexico unilaterally enjoys tariff-free access to Turkey’s market while Turkey cannot have equivalent access to the Mexican market. Turkey has therefore demanded that the EU begin negotiations for the upgrading of the Customs Union.

The EU, for its part, finds the current Customs Union unsatisfactory for solving bilateral trade problems with Turkey ( European Commission, 2016). The European Commission condemns Turkey for the fact that it ‘has introduced various trade and market access barriers which also affect European companies. A number of these measures are in breach of the Customs Union rules’ ( European Commission, 2017: 77–78). The Commission considers that such problems can largely be solved if both sides renew the Customs Union to remodel it as one that is more appropriate for current problems and realities. Johannes Hahn, the then Commissioner for European Neighbourhood Policy and Enlargement Negotiations, clearly states that a modernisation of the Customs Union would ‘bring big benefits for the EU’ ( Vytiska, 2018).

The EU’s response to the renewal of the Customs Union has developed in three stages: (i) the basic agreement between the European Commission and Turkey to modernise the Customs Union (May 2015) and the recommendation by the European Commission to start the negotiations (December 2016), (ii) the EU’s announcement not to continue to work towards the modernisation of the Customs Union until the situation within Turkey improves (June 2018), and (iii) the European Council’s decision to launch a positive political agenda with Turkey, including the modernisation of the Customs Union. The first stage was when Turkey and the European Commission agreed in principle in May 2015 to modernise and expand the existing Customs Union between the two parties ( European Commission, 2020b). This objective was reiterated in the ‘EU-Turkey Statement’ in March 2016, when both parties agreed to deal with the massive flood of migrants and asylum seekers mainly from Syria and other Middle Eastern states who risked their lives crossing the Mediterranean Sea, hoping to reach EU member states such as Germany and Sweden ( European Council, 2016). On 21 December 2016, the European Commission adopted the recommendation for a Council decision authorising the opening of negotiations with Turkey on an agreement for the extension of the scope of the bilateral preferential trade relationship and the modernisation of the Customs Union ( European Commission, 2020b). The main element of the recommendation was to further liberalise agricultural products and cover services, public procurement, and sustainable development. The proposal also included better dispute settlement mechanisms between the two. In addition to providing for a common external tariff for the products covered, the Customs Union foresaw Turkey aligning with the *acquis communautaire* in several essential internal market areas, notably concerning industrial standards. It was originally the intention of the Maltese Presidency to provide the Commission with the mandate within their term by the end of June 2017 ( Vesterbye & Akman, 2017: 8). However, several member states, including Germany, were strongly opposed to starting any new trade deals with Turkey. So far, there is no sign that the EU member states will give a mandate to the European Commission to begin the negotiation.

The second stage was the decision and announcement by the General Affairs Council of the EU on 26 June 2018. This claimed that ‘Turkey has been moving further away from the European Union’ and ‘Turkey’s accession negotiations have therefore effectively come to a standstill’; thus, the Council ruled out not only the opening of the new chapters of the accession negotiations but also further works towards the modernisation of the Customs Union ( Council of the European Union, 2018, para. 35). This policy was endorsed by the European Council on 28–29 June. Since that announcement, the approach of Brussels was that there were two conditions for the start of talks on the modernisation of the Customs Union and progress in the membership process: i) initiatives towards democratisation and improving rule of law and ii) greater alignment with the EU’s foreign policy towards third countries ( Adar *et al.,* 2020). However, this policy by the EU did not leverage a review of the domestic and international stance by the Erdoğan administration; as a result, the bilateral relations between the two reached a stalemate.

A slightly more positive development was seen in the third stage, when on 1 October 2020 the European Council agreed to launch ‘a positive political EU-Turkey agenda with a specific emphasis on the modernisation of the Customs Union’ ( European Council, 2020, para. 19). However, the latest Commission’s report on Turkey, which was published just a few days after the declaration of the European Council, contained negative assessments of Turkey’s political situation and foreign policy and did not include a means to restart the Customs Union negotiations ( European Commission, 2020b).

It should be noted, however, that both European and Turkish experts argue that it is destructive for the EU to block the overhaul of the Customs Union process since it would be ‘tantamount to eliminating the most promising avenue of engagement with Turkey aimed at fostering greater rule-based governance’ ( Ülgen, 2017; for similar assessment, also see Adar *et al*., 2020). Some even argue that upgrading the Customs Union would contribute to Turkey’s integration into the world economy ( Vesterbye & Akman, 2017: 9) and that a wider and deeper agreement could spill over into areas like the rule of law, freedoms, and the judiciary ( Vesterbye & Akman, 2017: 12). Having an updated and comprehensive trade deal is congruent with the global trade strategy that has been pursued by the EU in recent years. As it stands, however, many member states of the EU consider that starting negotiations to upgrade the Customs Union is tantamount to overlooking or tolerating the current authoritarian Turkish regime ( Adar *et al*., 2020). The upgrading of the Customs Union has therefore become yet another issue of discord between Turkey and the EU.

This means that the conclusion of the EU–Japan EPA could, in theory, have an inevitable negative impact on Turkey, no matter how welcoming it may appear to be. In the scenario in which the renewal of the EU–Turkey Customs Union does not happen in the near future, and Japan–Turkey EPA negotiations need more time to be concluded, the Japan–EU EPA could be yet another example of an arrangement between the EU and a third country harming the Turkish economy. Even though such damages can partially be covered by the bilateral efforts of Japan and Turkey, such endeavours would be less effective in the absence of the upgraded Customs Union between the EU and Turkey. It should also be noted that Japan does not seem to be aware of the negative impact that a Japan–EU EPA could have on Turkey.

## EU–Japan strategic partnership: underutilised

Although the EU designated Japan as one of its Strategic Partners in 2003 when the European Security Strategy was published—and although Japan and the EU have declared that they are partners that share principal values and norms—in-depth consultations regarding specific problems involving the EU and Japan have not been sufficiently conducted so far (for more positive views on EU-Japan relationship, see Tsuruoka, 2008; Tsuruoka, 2019; Tsuruoka, 2020). While I have pointed out elsewhere that Japan and the EU have largely been unsuccessful in discussing and dealing with the issues concerning security in East Asia ( Higashino, 2016), the same phenomenon can be observed for the issues of the non-EU European countries ^1^. Among the thorniest issues are the deterioration of democracy in Turkey, particularly after the attempted coup in July 2016, and how to (or rather whether or not to) continue Turkey’s EU accession process against this background. However, the *communiqués* and declarations after various high-level meetings and summits give no hint that the EU and Japan discussed the situation in Turkey in detail. This implies that, from the EU perspective, the democratic crisis in Turkey and deteriorating EU−Turkey relationship are ‘European’ problems, which are not to be shared or discussed with Japan. Japan, for its part, does not have any political will to bring these issues to the discussion table with the EU. Considering that the EU and Japan have concluded the SPA, whose core objective is to establish consensus in various fields of cooperation, based on their shared fundamental values such as democracy, rule of law, and human rights, it is desirable to engage in dialogue concerning delicate issues such as the political situation in Turkey. After the provisional application of the SPA was agreed for 1 February 2019, the Joint Committee under the SPA was held twice, on 25 March 2019 and 31 January 2020. In neither of these meetings, however, were issues concerning Turkey raised ( Ministry of Foreign Affairs of Japan, 2019; Ministry of Foreign Affairs of Japan, 2020).

One of the relatively new arrangements that has the potential to foster EU–Japan cooperation but that has not yet been fully utilised is the Partnership on Sustainable Connectivity and Quality Infrastructure (henceforth ‘Connectivity Partnership’) that was signed between the EU and Japan in September 2019 ( European External Action Service, 2019). This partnership aimed to facilitate cooperation between the EU and Japan, particularly on connectivity and infrastructure, including digital, transport, energy and people-to-people exchanges in regions such as ‘the Western Balkans, Eastern Europe, Central Asia, Indo-Pacific, as well as in Africa’ ( European External Action Service, 2019, para. 2). As Shinzo Abe, the then Prime Minister of Japan, reiterated in his keynote speech at the Europa Connectivity Forum, this partnership forms an important part of the framework of the Japan–EU SPA, which would enable both sides to work on international affairs with shared values and principles ( Abe, 2019). The document that inaugurates this partnership claims that both sides intend to promote ‘free, open, rules-based, fair, non-discriminatory and predictable regional and international trade and investment, transparent procurement practices, the ensuring of debt sustainability and the high standards of economic, fiscal, financial, social and environmental sustainability’. Furthermore, it was largely regarded as an approach to counter the Belt and Road Initiative (BRI) by China, which raised concerns about the so-called debt spirals in the countries in regions such as Central Asia and the Western Balkans ( Emmott, 2019; Heijmans, 2019; Söderberg, 2020). Therefore, the Connectivity Partnership between the EU and Japan should not be viewed simply as an economic project, but as a reaction to the sense that the growing influence of China is a threat.

There could have been at least two vectors of potential for this Connectivity Partnership to fortify the Japan–EU relationship in their approach to Turkey: first, by positioning Turkey as a beneficiary of this Partnership, along with the regions listed, such as the Western Balkans and Eastern Europe, and second, by bringing Turkey’s expertise and experience into the projects undertaken within the framework of the partnership, in such regions as Central Asia, where Turkey has a much longer history of commitment. However, as it stands, it is highly unlikely that these potentials will be explored by either the EU or Japan because many of the EU Member States would not accept the positioning of Turkey as a beneficiary of newly developed EU projects, not least because of Turkey’s democratic backslide and its assertiveness in its foreign policy (see above). As for Japan, the current priority of the Connectivity Partnership is to mobilise this new form of cooperation between the EU and Japan as early as possible and counter Chinese influence in the targeted regions. It is therefore unthinkable for Japan to raise any delicate issues, including the idea of liaison with Turkey, as it could hinder this nascent partnership.

## Turkey−Japan political relationship: *extant*

On the surface, the relationship between Japan and Turkey has been ‘virtually free of serious friction or controversies’ ( Pehlivanturk, 2011-12). Particularly in Japan, there has been a long-rooted discourse in which Turkey is sympathetic to Japan. Such discourse is based upon the history of mutual assistance between the two countries. This ranges from the tragedy of the *Ertugrul* frigate that sank off the coast of Kushimoto in 1890 to the 1985 operation by the Turkish government and Turkish Airlines to rescue around 200 Japanese nationals who were left behind in Tehran during airstrikes in the Iran−Iraq war. Even the experience of mutual assistance at the time of major earthquakes, such as those in Izmit in 1999 and Van in 2011 in Turkey, and the Great East Japan Earthquake in March 2011, have served as the basis of a deep friendship between Turkey and Japan ( Higashino, 2014). Furthermore, the Abe administration placed special emphasis on strengthening the economic ties with Turkey, beginning with the previously negotiated EPA between Turkey and Japan.

As it stands today, there are two distinct problems concerning the Turkey−Japan relationship. First, the current Turkey−Japan relationship is still heavily dependent on the discourses of the ‘past’. In other words, rather contradictorily, by overemphasising their historical ties, the two countries have not made a sufficiently serious endeavour to renew and strengthen their relationship in a more up-to-date form ^2^. What is lacking is an effort to establish a solid and constructive policy dialogue, concerning global issues, between the two. Thus, the previous talks between the Turkish and Japanese governments have focused almost exclusively on the EPA negotiations and other kinds of economic cooperation, rarely touching upon the more delicate political and diplomatic issues, including the recent deterioration of the EU−Turkey relationship. Even though the worsening relationship between the EU and Turkey is not beneficial to Japan, Japan has not developed an effective way to mitigate such tensions between its most significant partners.

Second, Japan does not necessarily share the same level of concern as other Western societies, namely within the EU as well as others, about the authoritarian trend of Turkish politics and the growing isolation of Turkey in world politics. Even though Erdoğan is now widely described as a ‘dictator’, or at least, one of the ‘illiberal leaders’ along with Vladimir V. Putin of Russia or Viktor Orban of Hungary ( Guriev & Treisman, 2015), the frequency with which such concerns are expressed and reported in the Japanese media is distinctly low. Behind this lack of serious concern about current Turkish politics are several intertwining elements. Discourses that describe Turkey as a precious long-term friend of Japan have tended to restrict negative reporting of Turkey. There is a widespread tendency to see Turkey as a ‘victim’ of ‘discriminatory’ Western attitudes (e.g., Naito, 2020). Turkey’s stalemated EU accession process is largely seen as, rather simplistically, a false promise and expression of anti-Islamic sentiment by European countries. According to such a view, the hardening of Erdoğan’s attitude towards the EU has been regarded with a certain level of sympathy. Inevitably, such understandings circumvent balanced analyses of the ongoing political oppression by the Erdoğan administration.

Furthermore, the timing of the notable destabilisation of Turkish politics coincided with important moments for the Turkey−Japan EPA (i.e., the publication of the Report of the Joint Study Group for a Turkey−Japan EPA in July 2013 or the fifth round of EPA negotiation in early 2016). The relevant events included: mass protests at the Gezi Park and the overreaction of the Erdoğan administration in early summer 2013; the attempted coup in July 2017; and the subsequent large-scale suspension of officials and academics who were alleged to have ‘Gülenist links’. The causal relationship between EPA-related developments and Japan’s reserved position towards the obvious worsening of the political situation in Turkey requires further detailed analysis. Still, we can assume that Japan was less motivated to problematise Turkish politics while the EPA negotiations were proceeding soundly.

## Turkey’s relationship with other regional organisations: destabilising effects

It has been widely discussed within Europe in recent years that, at least partly because of the deteriorating relationship between Turkey and the EU, Turkey is drifting farther from the Western community and is rapidly approaching non-Western international frameworks. One indication of this is Turkey becoming a founding member of the Asian Infrastructure and Investment Bank (AIIB) in January 2016. Turkey’s recent approach to join the SCO has also attracted interest and raised concerns ( Chaziza, 2016; Wang, 2016; also see Antonenko, 2007). This reflects Turkey’s rapidly deepening interests towards China ( Çolakoğlu, 2010; Çolakoğlu, 2013; Çolakoğlu, 2014; Çolakoğlu, 2015; Çolakoğlu, 2018) and approaching China-led international institutions can be interpreted as Turkey’s diplomatic endeavour to strengthen its relationship with China both bilaterally and multilaterally. Turkey had already become a ‘dialogue partner’ of the SCO, as the first NATO member and EU candidate to acquire such a position, and in recent years Erdoğan mentioned his intention to upgrade Turkey’s relationship with the SCO several times; this stirred speculation that Turkey will discard the long sought after path of joining the EU. For instance, Erdoğan repeatedly mentioned that Turkey should not be ‘fixated’ on the idea of joining the EU and could seek full membership in the SCO ( *Hürriyet Daily News*, 2016). Judging from his statement, ‘[i]f we get into the SCO, we will say good-bye to the European Union’ (cited in Pantucci & Petersen, 2013), Erdoğan seems to believe that membership in the SCO is an alternative to membership in the EU ( Keck, 2013) and that approaching the former does mean deviating from the latter ( *Hürriyet Daily News*, 2013). Such statements may well be regarded as a retaliation against the EU, which would not make any tangible advancement in terms of the accession process to the EU, or as a bargaining chip in talks with the West ( Gaspers, 2017). There is also the view that Turkey sees the SCO as a useful economic platform, ‘on which infrastructure projects are being implemented with the participation of China’ ( Stepanov, 2016), but not as an international organisation that Turkey can finally settle into instead of the EU. At the same time, however, the consistency that can be observed in the series of Erdoğan’s statements concerning potential SCO membership should not be neglected.

As it stands, Beijing has verbally welcomed Erdoğan’s positive remarks about the SCO ( Ministry of Foreign Affairs of the Peoples Republic of China, 2016), but it seems to maintain a reserved stance towards the potential for Turkish accession to the organisation ( Gaspers & Huotari, 2017). Russia has also been reserved, with Putin refraining from mentioning anything positive or negative about the potential Turkish accession to the SCO.

These developments suggest that it is unlikely that Turkey’s integration into the SCO will go beyond the current status of dialogue partner. Also, as many experts point out, the Turkey–China relationship is not without its problems. According to Selçuk Çolakoğlu, diplomatic relations between the two countries have soured because (i) China lost the competition over the Sinop nuclear power plant tender *vis-à-vis* the Japanese–French consortium in 2013, (ii) the Chinese government never declared the Kurdistan Workers’ Party (PKK) to be a terrorist organisation, (iii) considerable differences exist between the Turkish and Chinese governments in terms of their treatment of the Uyghur diaspora, and (iv) Beijing’s imposition of new visa restrictions on Turkish citizens as of February 2016 made it almost impossible for Turkish citizens to visit China ( Çolakoğlu, 2018). However, after the attempted coup in July 2016, the Turkish government has tried to intensify its relationship with China. Diplomatic as well as economic channels at various levels have been expanded, especially strengthening the relationship between the Communist Party of China (CPC) and the Justice and Development Party (AKP).

However, it should be noted that, even though the EU regards the realisation of Turkish accession to the EU as unlikely, Turkey’s quest for closer ties to the SCO is not a welcome or desirable development. It is largely regarded as destabilising for transatlantic security, and intelligence sharing among Turkey and the EU/NATO would become increasingly difficult. Erdoğan’s *rapprochement* with China coincides with the timing of the EU’s concerns towards China and its increasing international influence ( Godement & Vasselier, 2017). Japan also considers that Turkey’s closer ties to China and the SCO would be detrimental to the sound bilateral relationship between Japan and Turkey. Japan has experienced significant economic competition with China, particularly concerning infrastructure in South-East and South Asia, and Turkey’s approach to China would only fuel more rampant economic competition between Japan and China, caused by the very country with which Japan has enjoyed an exceptionally sound relationship for many years.

## Conclusion

This paper has highlighted several problems pertaining to the EU–Turkey–Japan triangle. The intertwining of the three sides of this triangle and problems pertaining to these dyadic relationships have not been explicitly discussed in previous research. This paper sought to explore how the strengthening and worsening of the relationship of one side inevitably affects the other two. It also emphasised that the strengthening of one side of this triangle does not automatically lead to the strengthening of the other two sides; for instance, as this paper showed, the strengthening of the EU–Japan relationship through the recent EPA could potentially have negative impacts on Turkey’s trade with third countries. Also, as discussed in this paper, having a new cooperation framework like the Japan–EU Connectivity Partnership may not, at least in the short term, necessarily lead to a taboo-free relationship and widen the scope of the issues to be discussed between the two. In contrast, having this nascent framework may well have a hidden impact at least on the behaviour of Japan, as it tends to avoid bringing in delicate issues such as Turkey’s political situation being discussed between the EU and Japan.

For the time being, it is not realistic to expect a rapid and drastic amelioration of the EU–Turkey relationship, when the possibility of halting accession negotiations has been hinted at by both sides. The relationship between Japan and the EU has largely been underutilised; further steps are needed to conduct substantial and constructive dialogues about how to anchor Turkey firmly in the norms of the rule of law and democracy. Relations between Turkey and Japan need considerable upgrading, and much effort is also necessary to go beyond the comfortable discourse of the extant ‘old friendship’ and to have a more substantial political and diplomatic relationship. This paper also analysed how Turkey’s recent endeavour to build closer ties with the SCO is regarded with suspicion by Japan and the EU.

Achieving all these aims is an ambitious task. If Japan intends to maintain a sound and constructive relationship with both the EU and Turkey, it will immediately face a considerable dilemma; as long as Japan considers itself one of the global actors that shares fundamental principles with the EU, it should not ignore the destabilisation of Turkish politics. However, considering the long-rooted discourses on historical ties with Turkey, it is highly unlikely that straightforward criticism of the Erdoğan administration would be a feasible option for Japan. Striking the best balance between the two will be a tightrope walk for Japanese diplomacy in the years to come.

## Data availability

No data are associated with this article.

## Footnotes

^1^ So far, Japan has been eager to raise issues such as threats provoked by North Korea and the conduct of China in the South and East China Seas. The EU has raised issues such as the Ukraine crisis and migration crisis.

^2^ Compare, for instance, the ‘Turkey-Japan relationship’ and ‘Turkey-China relationship’ webpages at the Ministry of Foreign Affairs of Turkey. The latter contains much more concrete detail concerning recent political and economic cooperation. See http://www.mfa.gov.tr/relations-between-turkey-and-japan.en.mfa and http://www.mfa.gov.tr/relations-between-turkey-and-china.en.mfa.
