# Supplementary material for: Relations between the EU, Turkey, and Japan: dissonances in the strategic triangle
Source: F1000Res. 2021 Mar 2;10:171. [Version 1] doi: 10.12688/f1000research.51085.1 (PMC7970432; doi:10.12688/f1000research.51085.1)
Supplement: Peer review history

# Peer review: Reviewer response for version 1

**DOI:** 10.5256/f1000research.54198.r80820

**Published:** 2021-03-22

**Authors:** Kohei Imai

**Affiliations:** 1 Institute of Developing Economies, Tokyo, Japan

This paper examined Turkey-EU, EU-Japan, Japan-Turkey relations well. And the item of triangular relations is an undeveloped field in International Relations. Therefore, this article has a good viewpoint.

 Meanwhile, this article should clarify the following questions for a better explanation:

- Please explain the reason of case selection (why did you choose Japan-Turkey-EU triangle relations as a case?).
- Three bilateral relations are not meaningfully connected to each other in this article. It is necessary that this article attempts to adopt frameworks of trilateral relations like “strategic triangle” or “fateful triangle”.
- This manuscript focused on Turkey-SCO relations. However, historically, Turkish decision makers have taken care of great powers and major institutions. So, this article should also examine Turkey’s diplomacy toward great powers and other regional organizations like NATO. In other words, it is necessary that this paper should discuss Turkey’s balancing behaviors.

Is the topic of the opinion article discussed accurately in the context of the current literature?

Yes

Are arguments sufficiently supported by evidence from the published literature?

Partly

Are all factual statements correct and adequately supported by citations?

Yes

Are the conclusions drawn balanced and justified on the basis of the presented arguments?

Partly

Reviewer Expertise:

International Relations, Turkish foreign policy, Middle East Studies

I confirm that I have read this submission and believe that I have an appropriate level of expertise to confirm that it is of an acceptable scientific standard.

# Peer review: Reviewer response for version 1

**DOI:** 10.5256/f1000research.54198.r80819

**Published:** 2021-03-17

**Authors:** Paul M. Bacon

**Affiliations:** 1 Waseda University, Tokyo, Japan

This was an enjoyable and interesting paper. It was well researched and written, and I learned a lot about three significant bilateral relationships. In my opinion, it is clear that the article deserves to be published, and to be published with minor revisions, that is to say 'Approved', using the F1000 terminology.

 The author offers sophisticated, clear and detailed analysis of three relationships: EU-Japan, EU-Turkey, and Turkey-Japan. The significance of each of the relationships is established, as are the problems and potentials. A helpful distinction is drawn between economic factors such as trade and connectivity, and political factors such as human rights and democracy, with the latter being correctly regarded as more problematic. The sources that the author uses are well-respected experts on each of the three relationships, and are also up to date, covering recent developments comprehensively. The author also uses governmental texts very well, correctly identifying the most relevant official political and economic statements and other communications, to explain the evolving relationships of each of the parties.

 One dimension of the paper that I would like see further explored in subsequent work, which is beyond the scope of this valuable paper, is the idea of a ‘strategic triangle’. This concept features in the title, the abstract and the conclusion of the paper under discussion, and is used effectively in this paper, but descriptively rather than analytically. This is fine. However, the concept has a rich history in both game theory and strategic analysis (see for example Tatu, 1970 ^1^; Brown, 1976 ^2^; Pillsbury, 1977 ^3^; Gottlieb, 1977 ^4^; Garrett, 1979 ^5^; Dittmer, 1981 ^6^; and Paul, 2019 ^7^), and can be explored in more detail.

 Dittmer (1981) for example, argues that:

 The preconditions for a triangular relationship are that each player recognize the strategic salience of the three principals, and the relationship between any two will be affected by each player's relationship to the third. Within the triangle, there are three distinct pattern dynamics: the *ménage à trois*, consisting of mutually positive relationships among all three; the stable marriage, consisting of a bilateral relationship excluding the third, and the romantic triangle, consisting of one pivot player playing off two suitors. 

 There are two dimensions to this definition; the first is the mutual recognition of strategic salience, the second is the type of dynamic among the three principals. This first begs the question to what extent the three principals in the paper recognize each other as strategically salient. The IR literature on strategic triangles has often focused on the most powerful states in the system: China/Russia/US in the context of the Cold War, and China/India/US in the emerging international context. So, in order to have ‘triangleness’ as an attribute, the three principals have to meet two criteria: to be objectively important in their own right, and to recognize each other as such.

 Does Turkey meet either criterion, both in terms of absolute or relational power? At one level, the cognitive premise of the paper appears to be that this triangular relationship is significant: why isn’t the obvious potential for a *ménage à trois* being realized? But the author doesn’t really explain why we should think of Turkey and Japan as being objectively important, other than both being in the G20, and could also say more about the relative significance of the economic relationships.

 In fact, the EU-Japan and EU-Turkey trade relationships are highly important, and of roughly equal significance, while the Turkey-Japan relationship is quite insignificant by comparison. According to the European Commission (2019, https://trade.ec.europa.eu/doclib/docs/2006/september/tradoc_122530.pdf), EU-Turkey trade volume was 138 billion Euros, and EU-Japan trade volume 124 billion Euros, compared to a volume of under 4 billion Euros for Japan-Turkey trade (Daily Sabah, 2019, https://www.dailysabah.com/business/2019/07/02/turkey-japan-economic-cooperation-more-important-in-times-of-trade-protectionism).

 In the main body of the paper the author explains in a detailed and sophisticated way what the obstacles are to deeper political cooperation and alignment between the three principals. She also explains expertly how timing has been a factor; how Turkey’s worst human rights abuses have taken place at sensitive times with regard to the EU-Japan negotiations, meaning that Japan elected to tread carefully and to prioritize the EU relationship. So at one level the paper invites us to share an initial premise that this triangular relationship is important, but then proceeds to explain forensically and expertly why it hasn’t fulfilled its potential, and that in fact the EU-Japan side of the triangle has been more important.

 We could therefore, logically, argue that there is no strategic triangle, because Turkey isn’t important enough. Or, we could say that Turkey is important, but that the relationship can be characterized as a ‘stable marriage’ between the EU and Japan, somewhat at the expense of Turkey, as outlined in the Dittmer quote above. If she wishes to pursue this second option in future work, the author could perhaps say more to establish why Turkey is potentially important, emphasizing the significance of the EU-Turkey trade relationship, and the strategic significance of Turkey as a civilizational swing state with a natural leadership role in Central Asia, an area of great significance to Japan and China (Huntington, 1993 ^8^). Although the author does talk about Turkey’s possible membership of the SCO, it is not really explained why that is important, or indeed why Turkey can be a highly significant player in the region even if it does not fully join the SCO.

 I do think that a case can be made that Turkey is highly important strategically and civilizationally, but the author doesn’t really set out this positive case. This argument needs to be made more systematically at the start of the discussion in subsequent work. This would then establish the credentials of Turkey as a legitimate part of an EU-Japan-Turkey strategic triangle, and this then allows us to identify a puzzle: that Turkey is potentially important strategically, but Japan has largely acquiesced in the EU’s practice of marginalizing Turkey, meaning that Turkey is the junior partner in the triangle.

 In short, I would suggest that the triangular premise and its attributes need to be explored in more detail in subsequent work. The triangular dimension lies at the heart of what makes this paper an important contribution. And the triangle concept, used analytically rather than descriptively, can shed still more light on this particular case, and in turn show the approach could be profitably used in other cases, for example in the analysis of EU-US-China, or EU-China-Japan. EU Studies scholarship on the Asian region has in the past arguably been a little one-dimensional, focusing bilaterally on the EU’s strategic partnerships, when it can be far richer and more profitable to take a triangular approach. For example, it is difficult to gain a meaningful understanding of the EU-Japan relationship, without understanding its relation to the EU-China relationship (see Berkofsky *et al.*, 2019 ^9^) for a useful exception to this general rule, which looks at EU-Japan relations *in the shadow* of China). I look forward to reading future work on these issues from the author, which build on the foundations in this valuable paper.

Is the topic of the opinion article discussed accurately in the context of the current literature?

Yes

Are arguments sufficiently supported by evidence from the published literature?

Yes

Are all factual statements correct and adequately supported by citations?

Yes

Are the conclusions drawn balanced and justified on the basis of the presented arguments?

Yes

Reviewer Expertise:

The European Union, EU-Japan relations, the EU as an Indo-Pacific actor, Brexit, human security.

I confirm that I have read this submission and believe that I have an appropriate level of expertise to confirm that it is of an acceptable scientific standard.

## References

[1] : The Great Power Triangle: Washington – Moscow – Peking. *Paris: Atlantic Institute*.1970; Reference source

[2] : Chinese Politics and American Policy: A New Look at the Triangle. *Foreign Policy*.1976; 10.2307/1147867 10.2307/1147867

[3] : Future Sino-American Security Ties: The View from Tokyo, Moscow, and Peking. *International Security*.1977;1(4) : 10.2307/2538627 10.2307/2538627

[4] : Chinese Foreign Policy Factionalism and the Origins of the Strategic Triangle. *Santa Monica: RAND R-1902-NA*.1977; Reference source

[5] : China Policy and the Strategic Triangle’, in Oye, K.A., Rothchild, D. and Lieber, R. eds., Eagle Entangled: U.S. Foreign Policy in a Complex World. *London and New York: Longman*.1979;228-264 Reference source

[6] : The Strategic Triangle: An Elementary Game-Theoretical Analysis. *World Politics*.1981;33(4) : 10.2307/2010133 485-515 10.2307/2010133

[7] : Theorizing India–US–China strategic triangle. *India Review*.2019;18(4) : 10.1080/14736489.2019.1662190 348-367 10.1080/14736489.2019.1662190

[8] : The Clash of Civilizations?. *Foreign Affairs*.1993;72(3) : 10.2307/20045621 10.2307/20045621

[9] : The EU–Japan Partnership in the Shadow of China: The Crisis of Liberalism. *Routledge, New York*.2019; Reference source

# Peer review: Reviewer response for version 1

**DOI:** 10.5256/f1000research.54198.r80816

**Published:** 2021-03-08

**Authors:** Selçuk Çolakoğlu

**Affiliations:** 1 The Turkish Center for Asia Pacific Studies, Ankara, Turkey

The manuscript is original in subject which analyzes the EU, Japan, and Turkey relations in the triangle concept. Although there are plenty of academic publications analyzing relations between the EU and Japan, the EU and Turkey, Japan and Turkey separately, triangular relationship analyses are exceedingly rare in the related literature.

 If the author clarifies the outcomes of the new development between Turkey and Japan, the paper will be more updated. As creating a “trust gab” issue between Ankara and Tokyo, it can be elaborated the Carlos Ghosn escape from Japan to Lebanon via Istanbul by a private Turkish jet company in December 2019. The Turkish criminal court just found guilty two pilots and one manager of the Turkish jet company in February 2021. However, the current outcome of Turkish legal investigation is far from being satisfactory for the Japanese government considering that the Carlos Ghosn escape was only possible with some high-level help from the Turkish government. The author should clarify whether the Ghosn case has the potential to poison the bilateral relations between Ankara and Tokyo.

 There are some corrections to some dates and others in the manuscript. The corrections are in bold formats:

- Page 3, paragraph 2, “… EU–Turkey Customs Union that has been in place since **1996** …”.
- Page 3 paragraph 4, …(e.g., EU–Japan and Japan– **Turkey**).
- Page 4, paragraph 3, At the G20 **Cannes** Summit in November 2011, …
- Page 7, paragraph 3, the attempted coup in July **2016**; and...

After doing these minor revisions/corrections, the paper can be indexed.

Is the topic of the opinion article discussed accurately in the context of the current literature?

Yes

Are arguments sufficiently supported by evidence from the published literature?

Yes

Are all factual statements correct and adequately supported by citations?

Yes

Are the conclusions drawn balanced and justified on the basis of the presented arguments?

Yes

Reviewer Expertise:

International relations, security studies, foreign policy, and global governance.

I confirm that I have read this submission and believe that I have an appropriate level of expertise to confirm that it is of an acceptable scientific standard.

# Response

**Published:** 2021-03-15

**Authors:** Atsuko Higashino

**Affiliations:** University of Tsukuba, Japan

Professor Çolakoğlu, thank you for your insightful comments.

 I agree with your kind suggestions for correction concerning dates and other issues and will make the necessary revisions.

 Your comments on the potential change in Japan’s perception of Turkey because of the Ghosn case are interesting and certainly worth mentioning in my paper. My current conclusion, however, is that the series of events concerning Ghosn will not affect Japan-Turkey relations significantly. Having read your comments, I consulted articles from:

1. Japanese newspapers,
2. Turkish newspapers (in English, online), and
3. International newspapers that covered the Ghosn case, particularly the recent judgement by the criminal court of Turkey.

In the context of your pointing out that the judgement could be considered unsatisfactory by the Japanese government, I consider that the way the issue was reported in (1) is particularly relevant for my paper.

 What struck me as noteworthy in all cases was the scarcity of media coverage concerning this incident. All the articles that I consulted reported the contents of the judgement by the Turkish court very briefly. Furthermore, none of the articles in category (1) implied any potential discord between Japan and Turkey due to this judgement[1].

 I predict that this case will not have a significant negative impact on the bilateral political relationship between Japan and Turkey for the following reasons;

- Japanese public perception about the case is that Ghosn himself is responsible. Some implicate the Lebanese authorities in his escape from Japan[2], but I found no articles that place the blame on the Turkish authorities.
- After the flight to Lebanon, the Japanese public gradually lost interest in the case. This could explain the scarcity of media coverage concerning the judgement by the Turkish criminal court.
- One exceptional example is the strong reaction of the Japanese government towards the Opinion adopted on 20 November 2020 by the Working Group on Arbitrary Detention of the United Nations, which concluded that the measures that had been applied to the defendant Carlos Ghosn by the Government of Japan had constituted arbitrary detention. The Japanese government deemed the Opinion ‘totally unacceptable’ and ‘not legally binding[3]’. Needless to say, this Opinion had nothing to do with the Turkish government.

As you mention, some — in Japan or Turkey — believe that Ghosn’s flight could have been possible only ‘with some high-level help from the Turkish government’; however, I could not find any media reports that blamed the Turkish government for this. I therefore consider that, rather than implying a potential crisis for their bilateral relationship, this silence by the Japanese media concerning potential involvement by Turkish authorities might even indicate the ‘resilience’ of the relationship. In the context of my article, it might be possible to assert that even the Japan-Turkey relationship is not easily affected by incidents of this kind. I will consider whether I should refer to the Ghosn case in my article. I reiterate my sincere gratitude for your kind comments, which have stimulated my thoughts on this issue.

 [1] See, for example, https://www.tokyo-np.co.jp/article/88034; https://news.yahoo.co.jp/articles/25b8d813f7198677e1e50e52337a59f843b45081

 [2] https://www.bbc.com/news/world-50964040

 [3] https://www.mofa.go.jp/press/release/press3e_000135.html
